# Invasive Pulmonary Aspergillosis with Disseminated Infection in Immunocompetent Patient

**DOI:** 10.1155/2016/7984032

**Published:** 2016-05-05

**Authors:** Gabriel Moreno-González, Antoni Ricart de Mesones, Rachid Tazi-Mezalek, Maria Teresa Marron-Moya, Antoni Rosell, Rafael Mañez

**Affiliations:** ^1^Intensive Care Unit, Bellvitge University Hospital, Feixa Llarga, S/N, Hospitalet de Llobregat, 08907 Barcelona, Spain; ^2^Bronchoscopy Department, Bellvitge University Hospital, Feixa Llarga, S/N, Hospitalet de Llobregat, 08907 Barcelona, Spain; ^3^Forensic Laboratory, Institute of Legal Medicine of Catalunya, Gran Via de les Corts Catalanes 111, 08075 Barcelona, Spain

## Abstract

Invasive pulmonary aspergillosis (IPA) is a rare pathology with increasing incidence mainly in critical care settings and recently in immunocompetent patients. The mortality of the disease is very high, regardless of an early diagnosis and aggressive treatment. Here, we report a case of a 56 yr old previously healthy woman who was found unconscious at home and admitted to the emergency room with mild respiratory insufficiency. In the first 24 hours she developed an acute respiratory failure with new radiographic infiltrates requiring Intensive Care Unit admission. A severe obstructive pattern with impossibility of ventilation because of bilateral atelectasis was observed, requiring emergent venovenous extracorporeal membrane oxygenator device insertion. Bronchoscopy revealed occlusion of main bronchi, demonstrating by biopsy an invasive infection by* Aspergillus fumigatus* and* A. flavus*. Despite an aggressive treatment and vital support the patient had a fatal outcome. The forensic study confirms the diagnosis of IPA but also revealed the presence of disseminated aspergillosis.

## 1. Case Presentation

**Figure 1:**
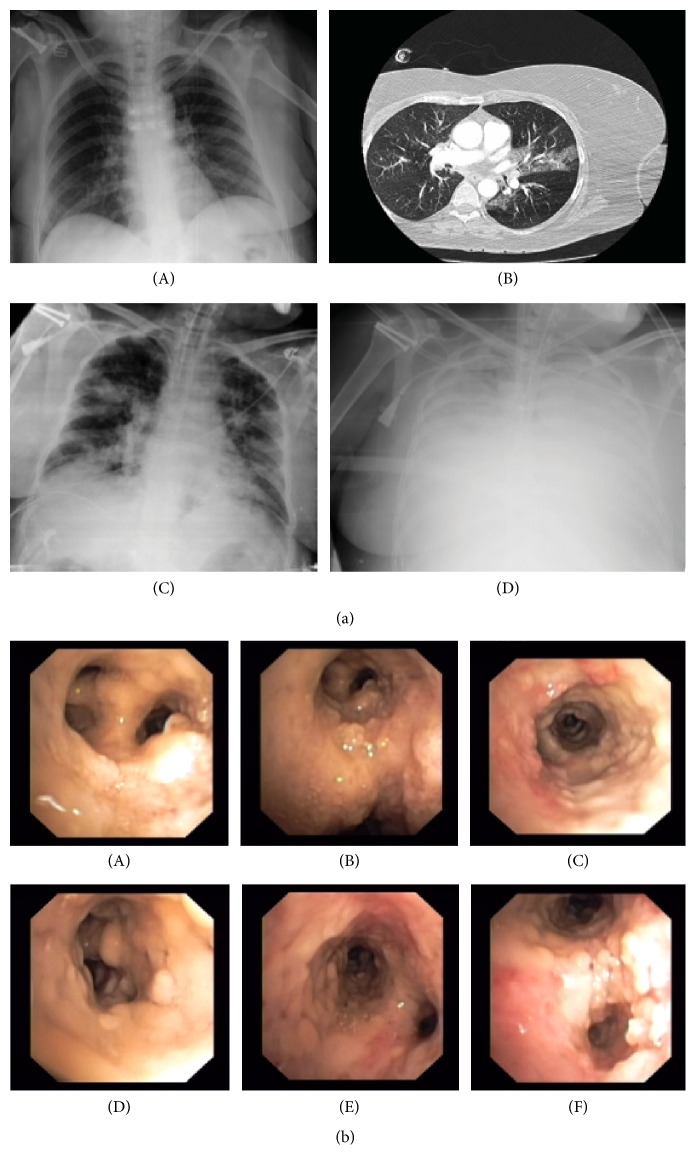
Radiological and bronchoscopic findings. (a)* Radiological findings.* (A) Chest radiography (CRx) at hospital admission (day 1). (B) Computed tomography revealing small lingula consolidation (day 2). (C) CRx at ICU admission with new bilateral infiltrates (day 3). (D) CRx after 24 h of ICU admittance showing bilateral atelectasis (day 4). (b)* Bronchoscopic findings.* (A) Necrotic detritus and pseudomembrane in the right upper lobe. (B) Right upper lobe and right carina with an extensive deposit of necrotic material. Bronchoscopic biopsy was performed and revealed the presence of fungus with hyphae suggestive of* Aspergillus*. (C) Involvement of intermediate bronchus. (D) The orifice of bronchus in the culmen was not visible because of a large quantity of necrotic material. Bronchial wash fluid revealed the presence of* Aspergillus*. (E) Necrotic detritus and pseudomembrane in the left upper lobe. (F) Involvement of culmen, left carina, and lingula.

A 56 yr old healthy woman was found at home in coma and with signs of long-standing immobilization such as pressure sores and dehydration. She had a history of 14-pack-year tobacco smoking and dyslipidemia treated with simvastatin 40 mg a day. There was no history of chronic pulmonary disease or other chronic diseases. Three days before admission she fell suffering mild head injury. The following day she was asymptomatic. After two days she was found in coma and delivered to the emergency room of our hospital. At hospital admission she had mild respiratory insufficiency with PaO_2_ : FIO_2_ ratio of 200 mmHg, bronchospasm, and mild depressed level of consciousness. Chest radiography was normal (Figure 1(a)(A)). Biochemical analysis at admission showed acute kidney injury with urea of 41 mmol/L (3.3–8 mmol/L), creatinine 120 *μ*mol/L (0–85 *μ*mol/L), and creatine phosphokinase of 15 mckat/L (0.05–2.3). The complete blood count showed hemoglobin of 16.6 g/L and 11,800 leucocytes, D-dimer of 680 mcg/L (<200), and normal clothing times. Brain computed tomography (CT) scan was performed and reported as normal and lumbar puncture revealed a normal cerebrospinal fluid. Thoracic CT scan ruled out pulmonary thromboembolism but a small lingula consolidation was observed (see Figure 1(a)(B)).

**Figure 2:**
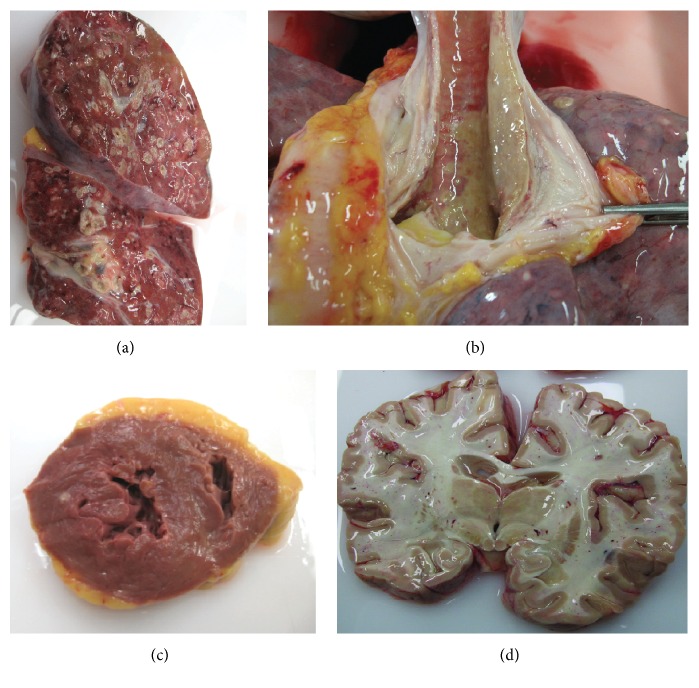
Pathological findings. (a) Lung. The image shows left and right lungs with bilateral necrotizing pneumonia with vascular invasion. Left lung weight: 875 gr. Right lung weight: 1040 gr. (b) Trachea. The trachea and main bronchi affected by invasive aspergillosis. (c) Heart. The white nodule in the left ventricle was reported as aspergilloma. (d) Brain. Several hemorrhagic and necrotic areas caused by septic microemboli of* Aspergillus*.

Initial management included fluid reanimation with crystalloids, bronchodilators, and empiric antibiotics (amoxicillin/clavulanic acid) as suspicion of aspiration pneumonia. However, 24 hours later she developed severe mixed acute respiratory failure with severe hypoxemia (pO_2_ : FIO_2_ of 100 mmHg) and respiratory acidosis with pCO_2_ of 73 mmHg associated with new radiographic infiltrates (Figure  1(a)(C)), requiring admission to the Intensive Care Unit (ICU), emergent orotracheal intubation, and initiation of invasive mechanical ventilation. A severe obstructive pattern was observed with impossibility of ventilation and chest radiography revealed complete bilateral infiltrates (Figure 1(a)(D)). The patient persisted with severe respiratory insufficiency. Prone positioning was not effective and an ECMO was inserted within the first 24 hr. of ICU admission. Fiber-optic bronchoscopy showed a large quantity of necrotic material at the distal bronchial tree, with impossibility of progress with the bronchoscope to subsegmental bronchi (Figure 1(b)). The serology was positive for galactomannan whereas histologic studies and culture of three different samples of the mucosa revealed* Aspergillus fumigatus* and* Aspergillus flavus*. We started intravenous voriconazole, caspofungin, and nebulized liposomal amphotericin. One week later the patient presented hemodynamic instability, worsening renal function and progressive multiorgan failure, and death. A forensic autopsy was performed showing invasive pulmonary aspergillosis (IPA) with disseminated infection involving lungs (bilateral necrotizing pneumonia), brain (necrotic and hemorrhagic spots suggesting septic emboli), heart, and kidneys (see Figure 2).

## 2. Discussion

The IPA is a rare pathology and the most devastating form of* Aspergillus* infection, characterized by invasion and necrosis of lung parenchyma [1]. There are several recognized risk factors to develop IPA, most of them related to different types of immunodeficiencies [2]. Classical risk factors associated with IPA include systemic or local radiotherapy, chemotherapy, airway constriction, prior inhaled (more than 2 weeks) or systemic (more than 3 weeks) corticosteroid use, hypoproteinaemia, prior antibiotic therapy (more than 2 weeks), neutropenia (less than 500 neutrophils/mm^3^ for more than 10 days), and chronic granulomatous diseases [3]. The risk of IPA correlates with the duration and degree of neutropenia and is higher following allogenic rather than autologous hematopoietic stem cell transplantation. Also, patients with severe graft versus host disease after hematopoietic stem cell transplantation, after solid organ transplantation, CMV lung infection, and CD4 count less than 100 cells/mm^3^ have a higher incidence of IPA [2]. In all of these conditions, IPA has a significant mortality ranging from 50% in neutropenic patients to 90% in hematopoietic stem cell transplantation patients [3, 4].

Patients admitted to ICU also showed an increased frequency of IPA (ranging from 0.33% to 6.9%) [3, 5]. Risk factors identified in ICU patients include chronic obstructive pulmonary disease, cirrhosis, solid organ transplantation, and severe sepsis [6].

Other factors associated with IPA in ICU include severe burns, short steroid treatment (less than 7 days), prolonged ICU stay (more than 21 days), malnutrition, and postcardiac surgery status [7]. Here, we report a previously healthy patient who developed IPA without any evidence of risk factors for this condition. The presence of IPA in nonimmunocompromised patients is growing [8–10]. Most of the reports are single cases initially treated with empiric antibiotics because of mild symptoms, some receiving antituberculosis treatment due to atypical presentation and high frequency of tuberculosis in the region, until the diagnosis of IPA [11, 12]. More than half of immunocompetent patients with invasive aspergillosis have underlying diseases (tuberculosis infection, diabetes mellitus, bronchiectasis, pulmonary sequestration, solid cancer, or chronic liver disease) [13]. However, to our knowledge dehydration and acute kidney injury, the only factors identified in this case, are not associated with IPA. In some cases IPA is related to previous colonization of the bronchial tree leading to epithelial damage and invasion [2] or to heavy inoculum of* Aspergillus* [10]. There is no evidence that any of these two circumstances affected our patient.

The diagnosis of IPA remains challenging and a high index of suspicion is necessary. A retrospective cohort study of nonneutropenic patients with (80%) or without (20%) underlying conditions and diagnosed with IPA demonstrated that clinical presentation (cough, dyspnea, fever, weight loss, hemoptysis, chest tightness, or chest pain) was similar [14]. Other studies suggest that hemoptysis is an important symptom present in around 60% of patients with IPA, but that in general, the clinical findings in patients without risk factors are nonspecific and do not aid in the diagnosis [13]. The typical radiological signs (presence of air crescent sign and the halo sign in CRx or CT scan) were not observed in any condition, whereas bronchoscopic findings and cultures were comparable [14]. In this case, the patient had wheezing, dyspnea, and severe respiratory insufficiency along with new radiological infiltrates nonsuggestive of IPA initially oriented as acute respiratory distress syndrome and aspiration pneumonia. The association of nonspecific clinical findings and the atypical radiological manifestations makes the diagnosis of IPA difficult and leads to a high misdiagnosis rate [13].

The histopathological examination of lung tissue by thoracoscopic or open-lung biopsy is the gold standard to diagnose IPA. Here, because of impossibility of progressing distally, we obtained a biopsy of the necrotic material in proximal bronchi, revealing the presence of two* Aspergillus* species. Mixed infection of different* Aspergillus* species is rarely reported in the literature [15] and associated with severe immunodepression and treatment failure, making our case even more exceptional.

Voriconazole 6 mg/kg IV b.i.d. for 1 day, followed by 4 mg/kg IV b.i.d., is the primary therapy for IPA [16]. We added caspofungin 70 mg in the first day and 50 mg the day thereafter due to the severity of the disease and the presence of two different* Aspergillus* species. Finally, we added nebulized liposomal amphotericin B to the treatment as an adjuvant therapy according to previous reports [17].

The risk factors associated with disseminated aspergillosis are the same for IPA. However, there are few cases reported in the literature in immunocompetent patients [9, 11, 12, 18]. In our case the patient had no relevant past medical history, the analysis did not reveal immunocompromise, tumoral markers were negative, and the pathological findings did not show any underlying disease besides the disseminated aspergillosis. So the presence of IPA with disseminated infection and the presence of two different* Aspergillus* species in the same patient were unexpected. Early diagnosis and correct aggressive treatment are associated with better outcome in patients with IPA [11]. However, although we initiated early appropriate aggressive treatment, the patient developed multiorgan failure and death revealing the severity of IPA in nonimmunocompromised patients.

## Additional Points

 Learning objectives are as follows:

- To have invasive pulmonary aspergillosis into the differential diagnostics in acute respiratory failure in emergency room or intensive care when more common etiologies are excluded and the patient has no adequate clinical response to antibiotic therapy.
- To understand that in rare cases severe invasive pulmonary aspergillosis with disseminated infection can affect immunocompetent patients.

*CanMEDS Competency: Medical Expert*

 Pretest questions are as follows:

1. Which are the frequency and mortality rates of invasive aspergillosis infection in immunocompetent patients admitted to an Intensive Care Unit?
2. How to diagnose invasive pulmonary aspergillosis in nonimmunocompromised patients?

Posttest questions are as follows:

1. Which are the frequency and mortality rates of invasive aspergillosis infection in immunocompetent patients admitted to an Intensive Care Unit?
   The incidence of invasive aspergillosis in ICU is around 0.33 but some studies report it as high as 6.9% in ICU population. The mortality rates are around 38% in colonized patients and 67% in putative invasive aspergillosis but can be as high as 80% (almost similar to the incidence in hematopoietic stem cell transplantation recipients).
2. How to diagnose invasive pulmonary aspergillosis in nonimmunocompromised patients?
   There is no specific clinical presentation of invasive pulmonary aspergillosis in immunocompetent patients. Also, the classical radiological signs in chest X-rays or computer tomography are absent. A high index of suspicion, bronchoscopy findings, microbiological sampling, and pulmonary biopsy are the main stem for diagnosis of IPA in immunocompetent patients.
